# A spatial analysis of human *Schistosoma japonicum* infections in Hubei, China, during 2009–2014

**DOI:** 10.1186/s13071-016-1817-6

**Published:** 2016-10-04

**Authors:** Hong Zhu, Shun-Xiang Cai, Jian-Bing Liu, Zu-Wu Tu, Jing Xia, Xiao-Wei Shan, Juan Qiu, Yong Jiang, Ying Xiao, Li Tang, Xi-Bao Huang

**Affiliations:** 1 Hubei Center for Disease Control and Prevention, Hubei Provincial Academy of Preventive Medicine, Wuhan, 430079 China; 2 Key Laboratory for Environment and Disaster Monitoring and Evaluation, Hubei, Institute of Geodesy and Geophysics, Chinese Academy of Sciences, 430077 Wuhan, China

**Keywords:** Spatial analysis, *Schistosoma japonicum*, Schistosomiasis, Hubei province

## Abstract

**Background:**

The province of Hubei is located in the middle of China, near the middle and lower reaches of the River Yangtze, and is an area where schistosomiasis is endemic. It is challenging to control this disease in this environment, and it would be useful to identify clusters of infection and transmission, as well as their distributions during recent years. Therefore, this study aimed to analyze the spatial distribution of schistosomiasis in Hubei, in order to facilitate the effective control and elimination of this disease.

**Methods:**

We collected schistosomiasis surveillance data from all endemic counties in Hubei during 2009–2014. A geographical information system (ArcGIS, version 10.1) was used to link the counties’ geographical data with the epidemiological data, and the spatial scanning method (FleXScan v3.1.2) was used to identify spatial clusters of human infections with *Schistosoma japonicum*.

**Results:**

In Hubei, patients who exhibited stool test results that were positive for *S. japonicum* accounted for > 50 % of all cases in China during 2009–2014. However, each endemic county in Hubei exhibited a declining trend in the number of human *S. japonicum* infections during the study period. The ArcGIS analyses revealed that the middle reaches of the River Yangtze were highly endemic for *S. japonicum* infections. Spatial scan analyses revealed the following infection clusters: two clusters in ten counties during 2009, two clusters in nine counties during 2010, three clusters in 12 counties during 2011, two clusters in 12 counties during both 2012 and 2013 and two clusters in ten counties during 2014. Most of the cluster regions were located in the lake and marshland regions along the basins of the River Yangtze.

**Conclusion:**

We successfully identified schistosomiasis clusters at the county level in Hubei during 2009–2014, and our results revealed that the clusters were typically located in lake and marshland regions. These data may be useful for controlling and eliminating schistosomiasis in other high-risk areas.

## Background

Schistosomiasis is caused by *Schistosoma japonicum*, and is one of the most serious parasitic diseases in the People’s Republic of China [1]. *S. japonicum* is transmitted by a snail intermediate host, which is a subspecies of *Oncomelania hupensis* (Gastropoda: Pomatiopsidae). Schistosomiasis has been endemic in 12 Chinese provinces along the River Yangtze, although significant progress has been made in controlling schistosomiasis since the mid-1950s [2]. Since 1985, five provinces and municipalities (Guangdong, Shanghai, Fujian, Guangxi, and Zhejiang) have subsequently fulfilled the criterion for transmission interruption [3]. Thus, schistosomiasis is mainly endemic in lake and marshland regions (located in the provinces of Hubei, Hunan, Jiangxi, Anhui, and Jiangsu) and in hilly and mountainous regions (located in the provinces of Sichuan and Yunnan) [4]. In 2004, a national medium-to-long-term strategic plan was launched to accelerate the process of schistosomiasis control throughout China [5]. Furthermore, an integrated strategy that emphasizes infection source control has been in place since 2005 [6], and the schistosomiasis epidemic in China exhibited a dramatic decline after these prevention and control measures were implemented [7].

**Fig. 1:**
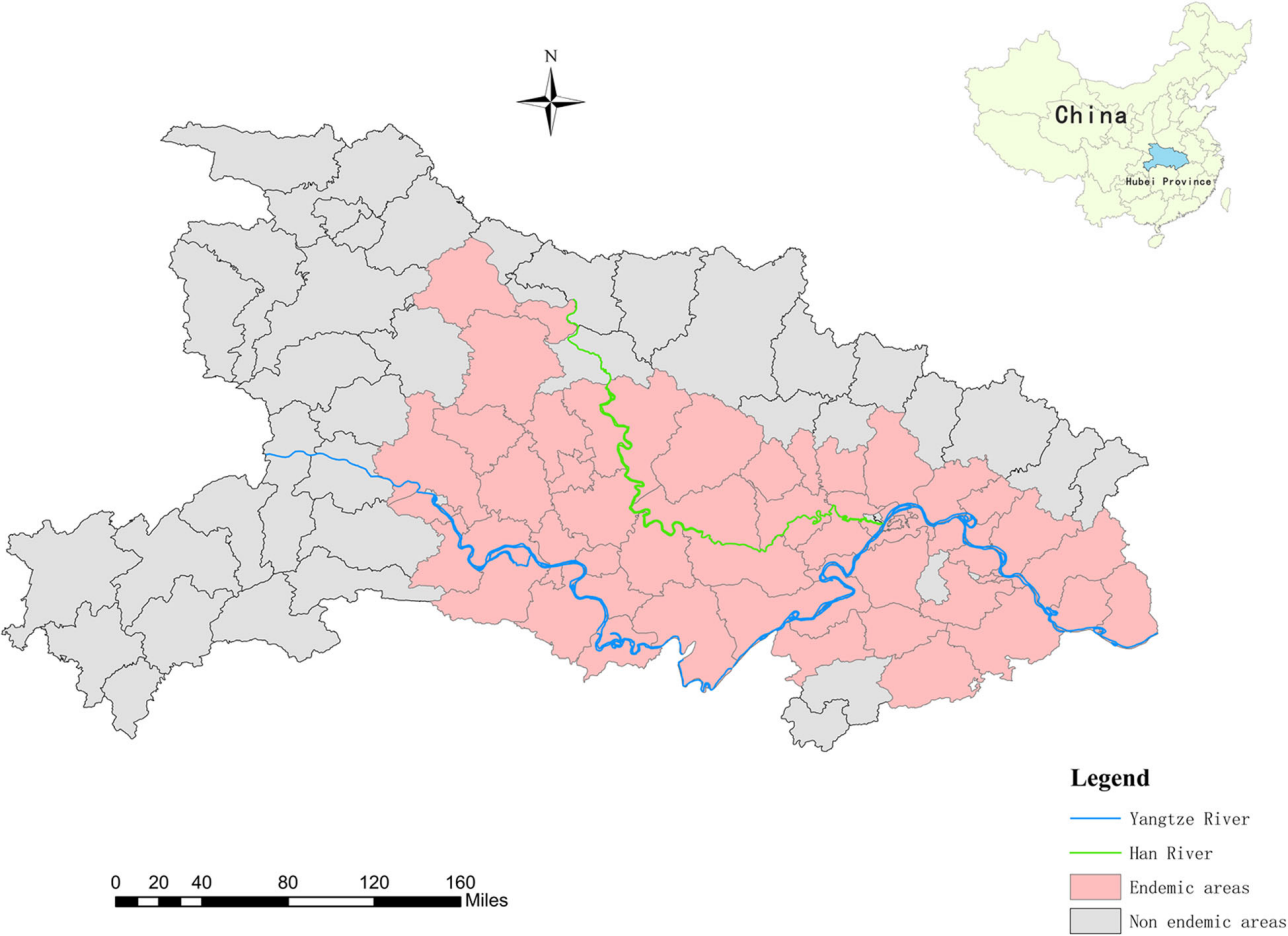
Map of endemic schistosomiasis in Hubei

The province of Hubei is located in the middle of China, near the middle and lower reaches of the River Yangtze (Fig. 1), and is one of the five lake and marshland regions where schistosomiasis remains endemic. *S. japonicum* has existed for over 2100 years in Hubei, and this fact has been confirmed by the presence of *S. japonicum* eggs in a corpse from the Western Han dynasty that was exhumed in 1975 (Jiangling Hsien, Hubei) [8]. In addition to being an endemic area, Hubei has one of the highest rates of schistosomiasis transmission in China [9]. However, the extensive control efforts during recent decades, and especially the integrated and comprehensive control measures that were implemented in 2006, helped Hubei fulfill the criterion for infection control in 2008 (i.e. a human/bovine prevalence of < 5 % in each village) [10]. Therefore, the medium-term goal of schistosomiasis control was successfully achieved in Hubei by the end of 2008 [4]. Subsequent efforts from the central and local governments have helped create a work plan to produce an effective control program. The Hubei government launched the new program (“Provinces and Ministries Union with the Ministry of Health and the Ministry of Agriculture”) in 2008, and this program was implemented in 2009 [11]. Between 2009 and 2013, the schistosomiasis control measures included replacing bovines with tractors for agricultural production, rearing domestic animals in pens, preventing domestic animals from roaming in the marshlands, recycling human and livestock excreta for biogas production, controlling *Oncomelania* species by promoting forestation and moving paddy fields to upland of high-risk areas, improving the water supply, enhancing sanitation and personal hygiene and other routine health-control measures (e.g. praziquantel treatment for humans and bovines, a snail survey and elimination and health education) [12–15]. In 2013, 5 years after this control program was implemented, all endemic counties in Hubei fulfilled the criterion for transmission control (i.e. a human/bovine prevalence of < 1 % in each village and the absence of snail infections). Thus, the long-term goal of schistosomiasis control in Hubei was achieved approximately 2 years ahead of schedule [16].

A few years after achieving schistosomiasis transmission control, the Hubei government attempted to achieve transmission interruption and the elimination of schistosomiasis [17]. However, there are several risk factors that have not been eliminated, and the reemergence of schistosomiasis is possible if efficient control measures are not enforced [18, 19]. Therefore, it is important to consolidate prevention and treatment efforts, and a new “Control and Elimination of Schistosomiasis” policy (to replace the 2006 policy) was published in 2015 and implemented on January 1, 2016. In this context, the human prevalence of schistosomiasis is an important indicator of transmission interruption and elimination [20], and a stool test is currently the gold standard for detecting and confirming schistosomiasis in humans [10].

The field of spatial analysis in the context of disease research has existed since the 1990s, and this field uses geographical information systems (GIS) to analyze geographical and epidemiological data for specific diseases [21–23]. The recent development of FleXScan software has also allowed researchers to detect clusters of various diseases [24, 25], including the early detection of disease outbreaks [26], cancer [27, 28] and hepatitis A [29]. However, few studies have reported performing a spatial analysis of schistosomiasis clustering using the flexible spatial scan statistic. Furthermore, only one study has performed a spatial analysis of schistosomiasis clustering in Hubei, and there is little information from after the province-wide fulfillment of the national criterion for transmission control in 2013. Therefore, the present study aimed to examine cases of stool test-confirmed *S. japonicum* infection in Hubei, and to use GIS (ArcGIS) and spatial scanning (FleXScan) techniques to analyze the spatial clustering of schistosomiasis at the county level. These data are intended to improve our understanding of the current status of schistosomiasis in the different areas of Hubei, and to provide useful information for the formulation and effective implementation of future control strategies.

## Methods

### Study areas

This study was performed in the province of Hubei (Fig. 1), which is characterized by a relatively wet environment during the summer and a drier environment during the winter. This environment is ideal for the survival of *Oncomelania hupensis,* which has significantly affected schistosomiasis transmission during recent decades [30]. Furthermore, the residents frequently come into contact with water harbouring infective parasite larvae during their activities of daily living. This study covered 63 endemic counties (Fig. 1) and 5450 endemic villages in Hubei during 2009–2014.

### Data collection

Annual surveys regarding schistosomiasis were performed in each village during the study period; the resulting data were reported to the townships, and final data regarding the numbers of infected individuals were available at the county level. This study targeted residents of each village who were 6–65 years old, and over 90 % of these residents were screened every September to November using the indirect hemagglutination assay [31, 32]. Stool samples were subsequently collected from over 90 % of the individuals with positive serological results, in order to perform the miracidium-hatching test [33, 34]. Cases of human *S. japonicum* infection were defined as patients who exhibited positive results from both the serological and stool tests. We used Microsoft Excel 2007 (Microsoft, Redmond, Washington, USA) to create a database of the surveillance data from the endemic counties during 2009–2014. Data regarding the total number of cases in China were extracted from the annual “Endemic status of schistosomiasis in the People’s Republic of China” reports for 2009–2014 [16, 35–39].

### Constructing the spatial database

The spatial database was created by extracting information regarding the cases of *S. japonicum* infection, although these data did not contain coordinate information. Therefore, we extracted each county’s coordinates from an electronic map of China (1:4,000,000) using ArcGIS software (version 10.1) (Esri, Redlands, California, USA), and the geographical data for the endemic counties were merged with the *S. japonicum* infection data. These steps created a spatial database of human *S. japonicum* infections in Hubei that were confirmed using the stool test.

### Spatial cluster analysis

FleXScan software and flexible scan statistics (v 3.1.2) [40] were used to detect irregularly shaped clusters. The data were extracted according to year, and four data files were prepared: a coordinate file, a matrix definition file, a case file and a population file. In the coordinate file, each data line from the schistosomiasis endemic areas in Hubei included the area’s name, latitudes and longitudes. In the matrix definition file, which was created using the Hubei provincial atlas, each region was connected to its adjacent regions to establish a spatial matrix of schistosomiasis endemic areas in Hubei. The frequency of disease and the population in each area were included in the case file and population file, respectively.

Based on the parameters of the flexible scan statistic, which allow a maximum number of 20 areas per window, we used 15 areas as the limit length of the cluster (default), and a Poisson distribution was used in the probability model. As with the spatial scan statistic, the alternative hypothesis was tested using a log-likelihood ratio (LLR) test and Monte Carlo replications, as well as the expected number of cases and relative risk (RR). Larger LLR values indicate stronger clustering. The RR was calculated for each statistically significant cluster, by comparing the risk inside the cluster with the risk outside the cluster.

For this study, the *P*-value of the test was based on the null distribution of the LLR statistic with a large number of Monte Carlo replications (*n* = 999), using the dataset that was generated under the null hypothesis. Differences were considered statistically significant at a *P*-value of < 0.05. The flexible spatial scan statistic is also able to locate secondary clusters that do not overlap with the most likely cluster but are still statistically significant. Based on the results of these analyses, we mapped the clustering boundaries using the flexible scan statistics [41].

### GIS mapping

The changes in the numbers of reported cases at the county level in Hubei were plotted using ArcGIS software according to year during 2009–2014. The annual numbers of reported cases at the county level were evaluated to assess the spatial distributions of *S. japonicum* infections. ArcGIS software was also used to visualize the space cluster regions in map.

## Results

### Endemic status of human *S. japonicum* infections in Hubei during 2009–2014

**Fig. 2:**
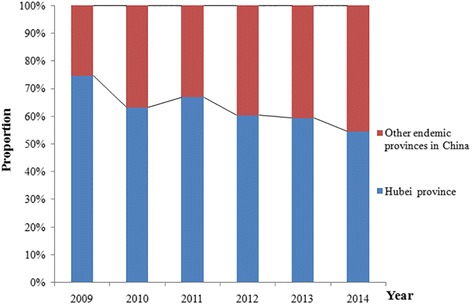
Changes in the proportions of *S. japonicum* infections in Hubei and China during 2009–2014

**Fig. 3:**
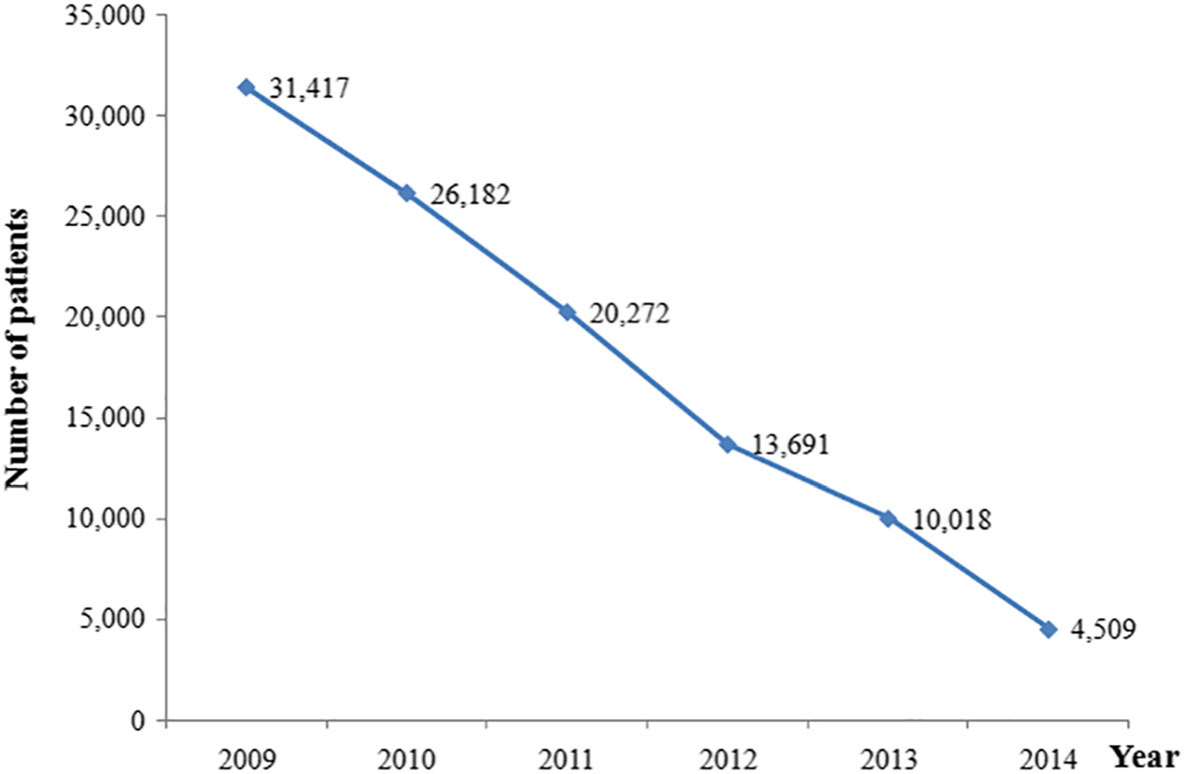
Annual trends in the incidence of *S. japonicum* infections in Hubei during 2009–2014

When we compared Hubei and the other endemic provinces in China, we found that Hubei accounted for 54.52–74.62 % of all Chinese cases during the study period (Fig. 2). Although this proportion exhibited a decreasing trend during the study period, Hubei accounted for > 50 % of all Chinese cases in all of the years that we evaluated. The stool test was used to confirm 31,417 cases of human *S. japonicum* infection in Hubei during 2009, and this number decreased to 4509 cases during 2014, which corresponded to a reduction of 85.65 % (Fig. 3). All cases from 2009 to 2014 could be classified to areas of infection control, transmission control and transmission interruption (Table 1).

**Table 1:** The cases of *S. japonicum* infections in different endemic types of Hubei during 2009–2014

Endemic types	Year					
	2009	2010	2011	2012	2013	2014
Infection control	30,291	25,163	19,423	13,026	9620	4509
Transmission control	1122	1016	849	664	398	0
Transmission interruption	4	3	0	1	0	0
Total	31,417	26,182	20,272	13,691	10,018	4509

### Spatial distribution of *S. japonicum* infections in Hubei

**Fig. 4:**
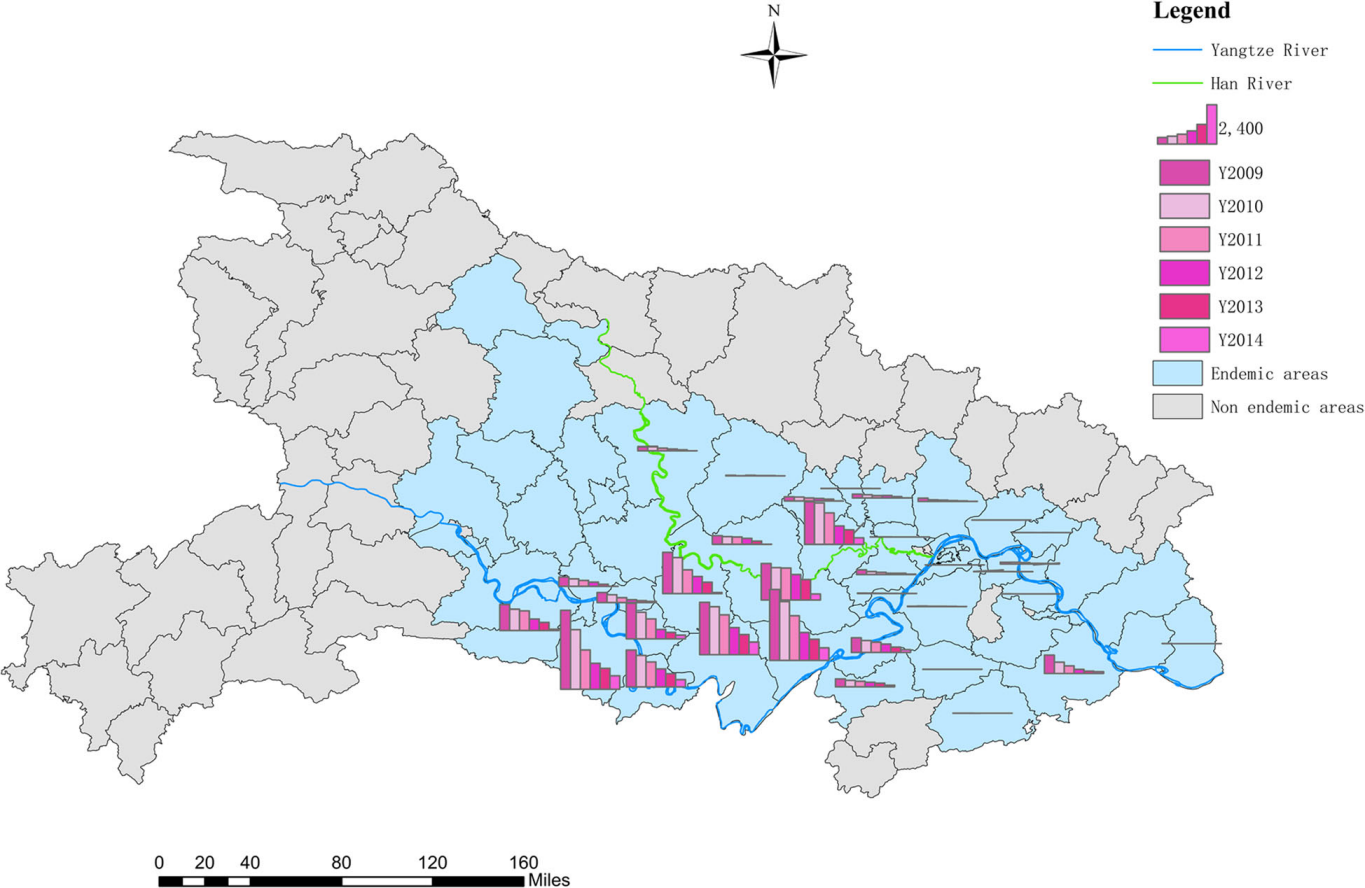
Spatial distribution of *S. japonicum* infections in Hubei during 2009–2014. *Abbreviation*: *Y* year

**Fig. 5:**
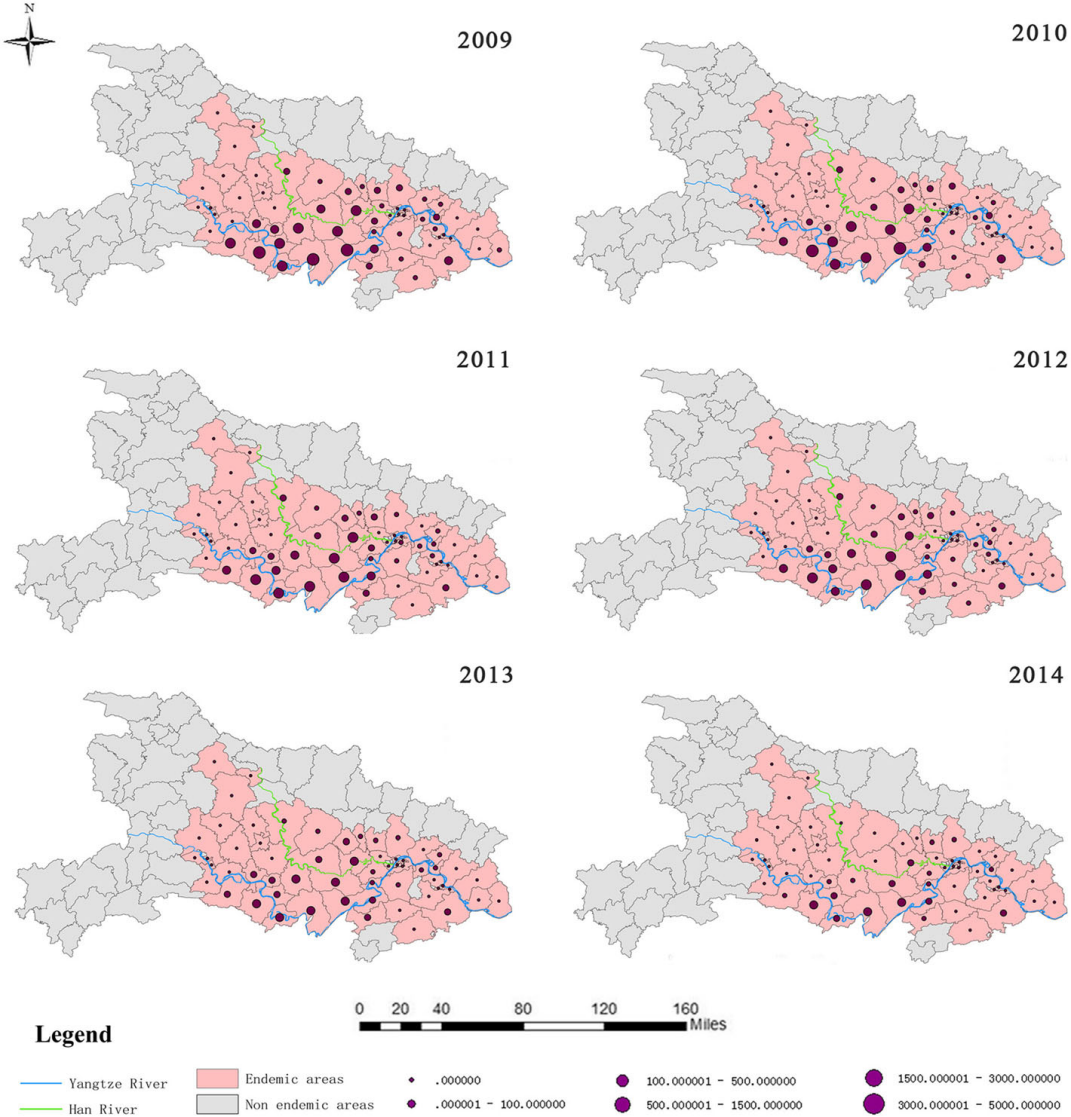
Changes in the spatial distributions of *S. japonicum* infections in Hubei during 2009–2014

Each endemic county in Hubei exhibited a declining trend in the incidences of human *S. japonicum* infections during 2009–2014. Counties that were highly endemic were typically located at the middle reaches of the rivers Yangtze and Han (Fig. 4). Among the 63 endemic counties in Hubei, > 500 confirmed cases of human *S. japonicum* infection were observed in 14 counties during 2009, in 11 counties during 2010, in 10 counties during 2011, in 10 counties during 2012, in 7 counties during 2013 and in 3 counties during 2014 (Fig. 5). For a more detailed stratification, the counties were classified as high-risk areas (3000–5000 cases), moderate-risk areas (1500–2999 cases) or low-risk areas (500–1499 cases). Figure 5 shows the changes in the counties’ classifications during the study period. We observed 0–3 high-risk areas, 0–6 moderate-risk areas and 3–7 low-risk areas during the study period.

### Spatial cluster analysis of *S. japonicum* infections in Hubei

**Fig. 6:**
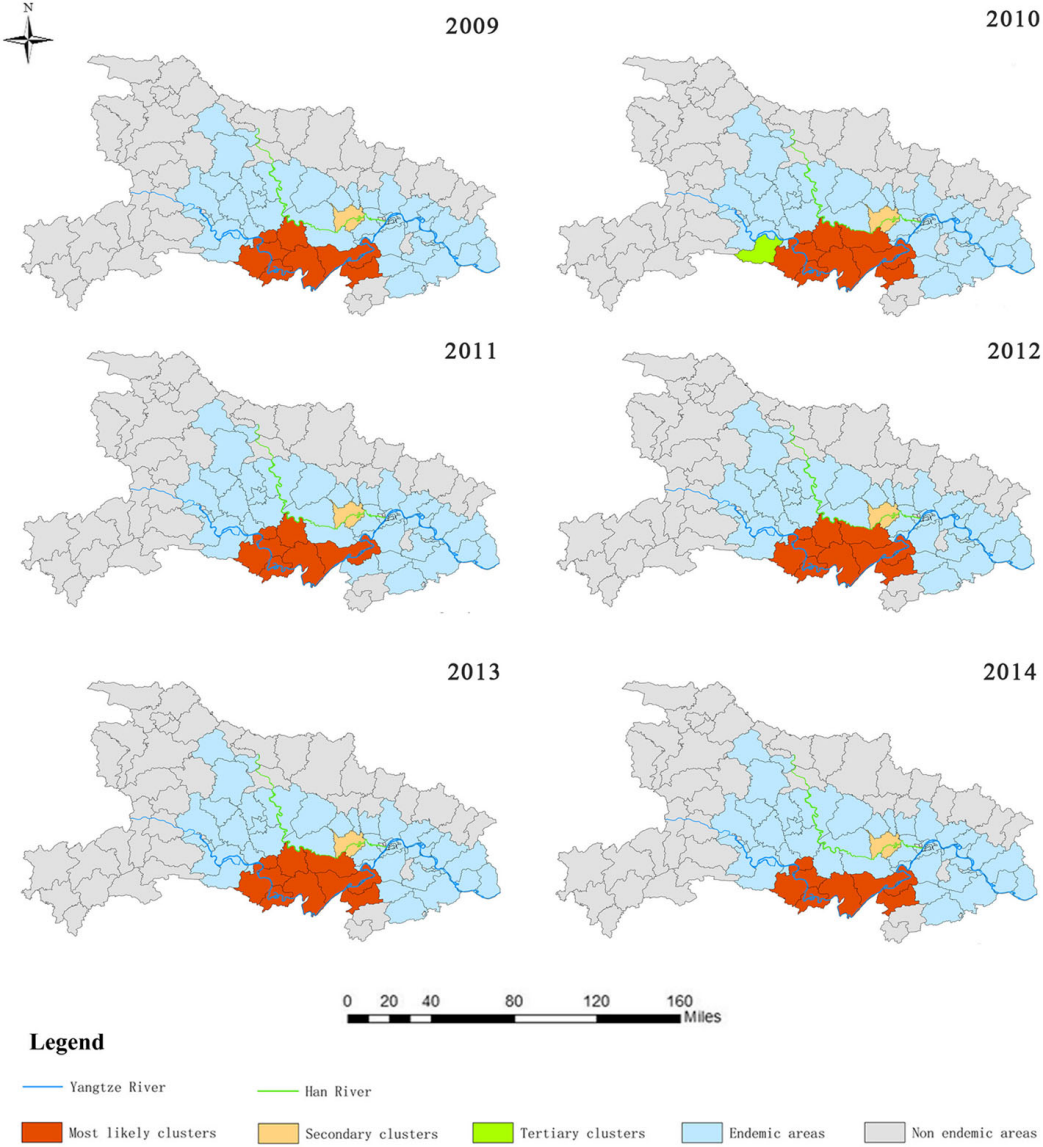
Clusters of *S. japonicum* infections in Hubei during 2009–2014

The cluster regions involved 10 counties during 2009, 9 counties during 2010, 12 counties during 2011–2013 and 10 counties during 2014. There was no difference in the 12 cluster regions between 2012 and 2013. Most of the cluster regions were located in lake and marshland regions along the basins of the River Yangtze (Fig. 6). There were three types of significant clusters: 6 most likely clusters, 6 secondary clusters, and 1 tertiary cluster (all *P* = 0.001). During 2009, the most likely cluster covered 9 counties and exhibited the greatest LLR value during the study period (LLR, 6254; RR, 1.85). The most likely cluster also covered 8 counties in 2009 and 2010, with the exception of Chibi (LLR, 4940; RR, 1.86) and 10 counties in 2011 (LLR, 3465; RR, 1.64). The only tertiary cluster appeared in 2011. In 2014, the most likely cluster covered 9 counties and exhibited the highest RR value during the study (RR, 2.49) (Table 2).

**Table 2:** The clusters of *S. japonicum* infections in Hubei during 2009–2014

Year	Types^a^	Reported cases	Expected cases	Log-likelihood ratio	Relative risk	*P-*value	No. of counties in cluster	Areas (County)
2009	1	21,224	11,443	6254	1.85	0.001	9	Shashi, Gong’an, Jianli, Jiangling, Shishou, Honghu, Jiayu, Chibi, Qianjiang
	2	2601	1657	244	1.57	0.001	1	Hanchuan
2010	1	17,072	9156	4940	1.86	0.001	8	Shashi, Gong’an, Jianli, Jiangling, Shishou, Honghu, Jiayu, Qianjiang
	2	2528	1381	408	1.83	0.001	1	Hanchuan
2011	1	14,938	9096	3465	1.64	0.001	10	Shashi, Gong’an, Jianli, Jiangling, Shishou, Honghu, Jiayu, Chibi, Xiantao, Qianjiang
	2	1921	1069	293	1.8	0.001	1	Hanchuan
	3	1189	1003	17	1.19	0.001	1	Songzi
2012	1	10,356	6243	2562	1.66	0.001	11	Shashi, Jingzhou Development Zone, Gong’an, Jianli, Jiangling, Shishou, Honghu, Jiayu, Chibi, Xiantao, Qianjiang
	2	1122	722	101	1.55	0.001	1	Hanchuan
2013	1	7737	4568	2090	1.69	0.001	11	Shashi, Jingzhou Development Zone, Gong’an, Jianli, Jiangling, Shishou, Honghu, Jiayu, Chibi, Xiantao, Qianjiang
	2	887	528	108	1.68	0.001	1	Hanchuan
2014	1	3445	1383	1998	2.49	0.001	9	Shashi, Jingzhou Development Zone, Gong’an, Jianli, Jiangling, Shishou, Honghu, Jiayu, Chibi
	2	402	238	50	1.69	0.001	1	Hanchuan

^a^1, most likely clusters; 2, secondary clusters; 3, tertiary clusters

## Discussion

Hubei has been an endemic hotspot for schistosomiasis, which may be related to its lake and marshland regions [42] and other biological and social factors that facilitate the transmission of *S. japonicum* [43]. In the present study, we found that Hubei accounted for >50 % of all human *S. japonicum* infections in China during 2009–2014, which indicates that better control strategies and measures are needed in Hubei. Furthermore, our spatial clustering data may provide useful information for controlling *S. japonicum* transmission in the lake and marshland regions of China.

During the study period, we observed a declining trend in all measures of *S. japonicum* infections in Hubei (e.g. in relation to all Chinese cases or in the absolute number of confirmed cases). In addition, almost all patients were located in areas that had achieved infection control and transmission control. These changes may be related to the annual surveillance of human infections, livestock infection, and infected snails, as well as the integrated infection control strategy that targeted control of the sources of infection in Hubei during 2009–2014. For example, the Provinces and Ministries Union program has covered 33 counties since the end of 2008 [44], and the “One Strategy One Village” program has covered 1442 heavily endemic villages since 2012 [45].

Our spatial analyses identified several areas in Hubei where schistosomiasis was endemic, and these areas were typically located along the middle reaches of the River Yangtze. These findings agree with the findings from previous studies that were performed in the provinces of Anhui and Jiangsu [46, 47]. Interestingly, we did not observe any major changes in the spatial distributions over time. This may be related to the fact that agricultural production and social habits in Hubei have not exhibited any major short-term changes, which would indicate that humans and animals still frequently come into contact with infected water, and that the resulting infections are difficult to control. We also used spatial scanning to evaluate clusters of infection during the study period, and our findings were similar to the spatial clustering of acute *S. japonicum* infections in China during 2005–2012 [48] and the high-risk areas that were identified before Hubei achieved schistosomiasis transmission control in 2013 [45]. In addition, although the total number of cases in 2009 (31,417) decreased to 4509 in 2014 (a reduction of 85.65 %), we still observed two clusters in 2014. These clusters are likely related to environmental risks in these regions, which contain a mixture of rivers, lakes and marshlands, as well as snails that serve as an infection vector (*Oncomelania hupensis*). Furthermore, the marshes typically dry up during the winter, and become flooded during the rainy season [49], and the large size of these areas makes it difficult to completely address these issues during a short period of time. Moreover, bovines are especially important to local agricultural production, and these animals can easily become infected in the marshland regions [50, 51]. These factors would indicate that residents and animals are still exposed to infection, because of their proximity to the River Yangtze and its branches. Therefore, control efforts should likely target these cluster areas, and new techniques and methods should be used to address the epidemic in these areas.

The present study includes several strengths. First, our analysis is based on absolute quantities, rather than the relative indicators of infection that have been used in previous studies (e.g. the infection rate or positive rate). Secondly, our spatial analysis using the flexible spatial scan statistic allowed us to identify infection clusters, which will be useful for directing future efforts to control schistosomiasis. However, this study also involves several limitations. First, our analyses were limited by the absence of detailed demographic data (e.g. sex, age and occupation), which precludes any adjustment for potentially confounding factors. Secondly, we only evaluated the incidences of *S. japonicum* infection at the county level, and additional studies are needed to provide higher-resolution spatial distributions (e.g. village or individual levels).

## Conclusion

In the present study, we identified county-based clusters of human *S. japonicum* infections in Hubei during 2009–2014, using positive stool test results and FleXScan software. These results indicate that control efforts should be focused on the middle reaches of the River Yangtze and its connecting branches. Given the importance of accurate epidemiological data regarding schistosomiasis, our results suggest that it may be useful to create a database of patients who exhibit positive stool test results, in order to facilitate dynamic monitoring of schistosomiasis.

## Abbreviations

GIS: Geographical Information Systems
LLR: Log-likelihood ratio
RR: Relative risk
